# The enhancement of emotional skills as a resource to reduce hopelessness

**DOI:** 10.1192/j.eurpsy.2021.2016

**Published:** 2021-08-13

**Authors:** I. Delhom, J.C. Melendez, E. Satorres

**Affiliations:** 1 Psychology, Valencian International University, Valencia, Spain; 2 Development Psychology, University of Valencia, Valencia, Spain; 3 Developmental Psychology, University of Valencia, Valencia, Spain

**Keywords:** Emotional intelligence, Hopelessness, mental health

## Abstract

**Introduction:**

Emotional Intelligence (EI) involves a set of emotional skills (attention, clarity, and emotional regulation) for the effective use of emotional information (Mayer & Salovey, 1997). The lack of emotional skills has been associated with multiple disruptive emotional phenomena, such as hopelessness. It has been observed that EI can be a predictor of hopelessness in older adults, in such a way that we can consider that the development of EI could be a relevant resource for promoting mental health in older adults.

**Objectives:**

Implement an EI intervention to reduce levels of hopelessness.

**Methods:**

The sample consisted of 123 healthy older adults from Valencia (Spain), randomly distributed into two groups: treatment group (N = 57) and control group (N = 68), aged between 60 and 84 years, with a mean age of 67.62 years (SD = 6.43). Of these, 54.4% were women and the remaining 45.6% were men. The Trait Meta-Mood Sclae 24 (TMMS 24) was applied to assess EI and the Beck Hopelessness Scale (BHS) to assess hopelessness.

**Results:**

Significant differences are observed in the treatment group after the intervention (F1, 123 = 19.86; p < 0.001, h2 = 0.142), with a decrease in scores (T1= 4.72; T2=2.88). For the control group, the effects were not significant (F1, 123 = 1.06; p = 0.305, h2 = 0,009).

**Conclusions:**

The efficacy of the intervention in EI to manage emotional states is evidenced, reducing levels of hopelessness thanks to training in adaptive emotional processing and emotional management skills.

**Disclosure:**

No significant relationships.

